# The General Practitioner and the Optometrist

**Published:** 1911-08

**Authors:** 


### The General Practitioner and the Optometrist.

MANY optometrists apply directly to physicians for the reference of refractive cases and, from the confidence with which they speak and the blanks which they have prepared, as well as from direct statements by both optometrists and physicians, we may conclude that such relations exist in considerable number.

Passing the natural hesitancy of the regularly graduated physician in special practice, to canvass the profession for reference of cases, it must be acknowledged that the proposition of the optometrist is fair and business like. It should be noted, however, that the request of the optometrist is very different from that of the manufacturing optician for prescriptions, which latter are fairly comparable to prescriptions for drugs or orders for instruments, prosthetic devices, and the like.

The optometrist bids for a branch of medical practice in active competition with the more modest, implied readiness to take similar cases, on the part of the doctor in medicine who has qualified as a specialist in diseases of the eye. The osteopath, as well as the extra-professional practitioner of various other kinds also competes with the regular physician, general practitioner or specialist, but he does so independently and without asking favors of the medical profession.

We raise the question whether, on either economic or ethical grounds, a member of the medical profession should aid in competition against his own profession. The fact that an individual practitioner does not himself perform the particular professional service in question or that the competitive process does not directly affect the man asked to refer cases but only a limited and specialised part of the profession, should influence our decision in no way. The ethical specialist is a bona fide member of the whole profession, participating in all its general interests, performing on the average a disproportionate share of the duty of the profession to care for its own members, contributing like every other physician to the strength of medical organisation and more than most to the general fund of knowledge, of the profession. He competes to a less degree than the average and less directly, with the general practitioner. Should the general practitioner or a specialist of another kind aid in extra-professional competition against a brother physician?

From the very narrow and immediate standpoint, the reference of cases to an optometrist, a midwife, an osteopath or any other extra-professional practitioner in a narrow field, may serve the interests of the physician who does so. But it is difficult to see how this can be so, except by the payment of commissions, frankly or in a round-about way and, on such methods the medical profession has expressed itself clearly and emphatically.

Even from an equally selfish but farther sighted view, it is clear that the usurpation of medical practice by extra-professional competitors, affords a precedent for the seizing of one detail after another which now helps support and train the entire profession. Successful competition with any specialist will simply tend to drive him back into general practice so that the fact that the physician who abets the efforts of the outside man to get medical practice does not immediately reduce his own practice, has no bearing on the ultimate result.

There is one more fact to be considered, in the interests of the patient. The eye is something more than an optic apparatus and the correction of visual defects demands much more than the measurements of curved surfaces. The ordinary run of eye cases may, perhaps, be treated by the simple application of principles of refraction. So the ordinary run of fracture cases might be treated by an ignorant bone setter with a good eye and a delicate tactile and muscular sense. Stomachs can be washed by a trained nurse, or by the patient himself, midwives can deliver the normal parous woman, almost anyone of average intelligence can be taught to pass the catheter, in fact, to perform any other detail of medical practice. But we must not forget the need of a guiding, broadly trained, medical intelligence that can not only perform the obvious mechanic detail but that can supervise the interests of the whole patient and that can judge correctly whether the case is merely one of mechanic detail or whether some serious general pathologic process underlies the particular manifestation.

